# Integrated Metabolome and Transcriptome Analysis of Gibberellins Mediated the Circadian Rhythm of Leaf Elongation by Regulating Lignin Synthesis in Maize

**DOI:** 10.3390/ijms25052705

**Published:** 2024-02-26

**Authors:** Qingqing Yao, Ying Feng, Jiajie Wang, Yushi Zhang, Fei Yi, Zhaohu Li, Mingcai Zhang

**Affiliations:** State Key Laboratory of Plant Environmental Resilience, Engineering Research Center of Plant Growth Regulator, Ministry of Education, College of Agronomy and Biotechnology, China Agricultural University, No 2 Yuanmingyuan West Road, Haidian District, Beijing 100193, China; ksy18801291882@163.com (Q.Y.); fyfengying1998@163.com (Y.F.); wjj19971010@126.com (J.W.); zhangyushi@cau.edu.cn (Y.Z.); yifei56@cau.edu.cn (F.Y.); lizhaohu@cau.edu.cn (Z.L.)

**Keywords:** maize (*Zea mays* L.), gibberellins, leaf elongation, cell wall, lignin, circadian genes

## Abstract

Plant growth exhibits rhythmic characteristics, and gibberellins (GAs) are involved in regulating cell growth, but it is still unclear how GAs crosstalk with circadian rhythm to regulate cell elongation. The study analyzed growth characteristics of wild-type (WT), *zmga3ox* and *zmga3ox* with GA_3_ seedlings. We integrated metabolomes and transcriptomes to study the interaction between GAs and circadian rhythm in mediating leaf elongation. The rates of leaf growth were higher in WT than *zmga3ox*, and *zmga3ox* cell length was shorter when proliferated in darkness than light, and GA_3_ restored *zmga3ox* leaf growth. The differentially expressed genes (DEGs) between WT and *zmga3ox* were mainly enriched in hormone signaling and cell wall synthesis, while DEGs in *zmga3ox* were restored to WT by GA_3_. Moreover, the number of circadian DEGs that reached the peak expression in darkness was more than light, and the upregulated circadian DEGs were mainly enriched in cell wall synthesis. The differentially accumulated metabolites (DAMs) were mainly attributed to flavonoids and phenolic acid. Twenty-two DAMs showed rhythmic accumulation, especially enriched in lignin synthesis. The circadian DEGs *ZmMYBr41/87* and *ZmHB34/70* were identified as regulators of *ZmHCT8* and *ZmBM1*, which were enzymes in lignin synthesis. Furthermore, GAs regulated *ZmMYBr41/87* and *ZmHB34/70* to modulate lignin biosynthesis for mediating leaf rhythmic growth.

## 1. Introduction

Gibberellins (GAs) are phytohormones of diterpenoids closely related to plant growth and development. GAs have been extensively studied for their regulation of plant type, and dwarf breeding can be achieved by improving the genes involved in GA synthesis and the signaling pathway [1,2,3,4,5,6]. Moreover, cell shape is mainly attributed to the formation of the primary and secondary cell wall, and the secondary cell wall is the layer of the cell wall that accumulates inside the primary cell wall after the cell stops growing [7]. Meanwhile, GAs can modulate cell wall relaxation and the biosynthesis process to control cell expansion, while the cell wall synthesis genes involved in cellulose, hemicellulose and lignin components are markedly regulated by GAs [8,9]. However, it is necessary to further explore how GAs promote cell growth.

Cell elongation has regular characteristics for controlling plant morphological characteristics. Previous studies have shown that hypocotyl elongation is controlled by the circadian rhythm system in *Arabidopsis* [10,11,12]. Moreover, this process is also related to photoperiodic changes, regulating hypocotyl growth through the interaction of the circadian rhythm with photoperiodic signals. Meanwhile, the cell wall construction process also exhibits a circadian rhythm, and the cell wall construction process is regulated by the circadian rhythm in maize [13]. During this process, cellulose and lignin synthase genes display circadian expression patterns, and the lignin synthesis genes, such as C4H1, caffeate O-methyltransferase (COMT) caffeoyl-CoA O-methyltransferase (CCoAOMT1), CCR1, and CAD6, exhibit circadian fluctuations at the transcriptional level [14]. This indicates the expansion and synthesis of cell walls are controlled by the circadian rhythm system. In addition, GAs have been reported to mediate the rhythmic expression of circadian genes associated with cell wall modification [15]. However, it is still unclear how GA signals crosstalk with circadian rhythm signals to regulate cell elongation in GA-induced plant growth.

The circadian rhythm acts as an internal timekeeper involved in regulating almost all growth, development and metabolic processes in plants [16,17,18,19,20,21]. The main function is to synchronize the internal physiological processes and coordinate metabolic activities by anticipating cyclic changes in the external environment [22]. The circadian proteins are highly expressed at distinct times during light and darkness, and their expression is regulated reciprocally at both the transcriptional and post-transcriptional levels. For instance, the circadian clock associated 1 (CCA1) and late elongated hypocotyl (LHY) genes are highly expressed in the morning and are inhibited during the expression of CAB expression 1 (TOC1). However, it is inhibited by TOC1 in the evening, and TOC1 expression peaks in the evening [23,24]. The pseudo-response regulator (PRR) gene family members are also involved in regulating CCA1 and LHY1 expression to ensure accurate morning high expression [25]. The circadian rhythm can interact with phytohormone signaling to influence plant growth or respond to stress. The PRR7 target is ABA DEFICIENT 1 encoding a zeaxanthin epoxidase involved in ABA biosynthesis, and ABA affects TOC1 expression, while LHY promotes the expression of ABA-responsive genes responsible for increased tolerance to drought and osmotic stress [26,27,28,29]. Moreover, the JA signals have demonstrated that the signal transduction elements, MYC2 or JAZ, interact with the circadian signaling element in response to the circadian rhythm [30,31,32], and the circadian signaling element can also regulate the expression of JA synthesis genes [33,34]. The circadian systems can also affect the expression of the ethylene synthetase ACC and ACS genes and ethylene responsive factor (ERF) [35,36,37], and ethylene may shorten the circadian period [38]. This suggests that plant hormones can act as a relay mechanism to modulate the amplitude and the phase of output rhythms.

The GA signal oscillation has been found to participate in circadian signaling networks, and GA receptors are controlled by circadian rhythms [15]. The DELLA proteins (DELLAs) are the key negative regulator of the GA signal [39], and GA signals regulate the expression of the hypocotyl elongation gene mainly through the crosstalk between DELLAs and circadian genes [40,41]. However, the mechanisms by which GAs regulate the expression of circadian genes affecting leaf cell growth have been rarely investigated. Cell growth is paralleled by cell wall expansion and synthesis, and GAs are known to mediate the regular expression of circadian genes associated with cell wall modification [15]. However, it was unclear how GA regulated the expression of circadian genes associated with cell wall synthesis for mediating rhythmic cell growth.

The aim of this study was to investigate the biological mechanism of GAs that interacted with circadian signals for regulating cell growth in the GA-mediated rhythmic growth of maize leaf. Here, the growth characteristics were analyzed in WT and *zmga3ox* (GAs biosynthesis mutant) and *zmga3ox* with GA_3_ seedlings. Then, integrated metabolome and transcriptome analysis was used to obtain the rhythmic expression of transcription factors and function genes regulated by GAs in the GA-mediated rhythmic leaf growth. Combined with gene regulatory network analysis (GRN), cis motif analysis, and dual-luciferase assay, the proposed working model was established to clarify the transcriptional regulatory pathway of GA-mediated rhythmic leaf growth. This study would provide new insights into the transcriptional regulation of GA-mediated rhythmic growth in maize. It also revealed rich genetic resources for improving GA signaling to achieve dwarfing and increase grain yield, and supplied a theoretical basis for the application of GAs in production practice. In addition, the study of leaf growth will facilitate the capture and utilization of photosynthetic energy.

## 2. Results

### 2.1. GA-Modulated Cell Elongation Involved in Regulating the Circadian Rhythm of Leaf Growth

**Figure 1:**
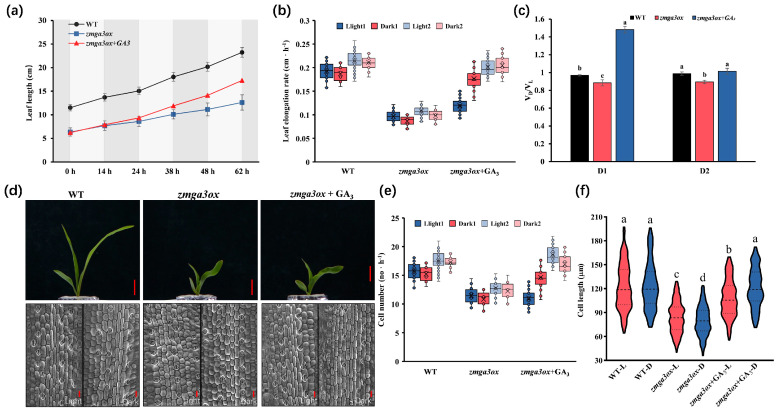
Characterization and phenotypic analysis of leaf growth in *zmga3ox*, *zmga3ox* + GA_3_ and WT seedlings. (**a**) The dynamic characteristics of leaf length of *zmga3ox*, *zmga3ox +* GA_3_ and WT seedlings. (**b**) The elongation rate of leaf length of *zmga3ox*, *zmga3ox +* GA_3_ and WT seedlings within 2 days. Light1 indicates the seedlings cultured under light for 0 to14 h; Dark1 indicates the seedlings cultured under darkness for 15 to 24 h; Light2 indicates the seedlings cultured under light for 25 to 36 h; Dark2 indicates the seedlings cultured under darkness for 37 to 48 h. Data were presented as mean  ±  SD (n  =  30). (**c**) The V_D_/V_L_ of *zmga3ox*, *zmga3ox +* GA_3_ and WT seedlings under D1 and D2. V_D_/V_L_ represented the ratio of leaf elongation rate of *zmga3ox*, *zmga3ox +* GA_3_ and WT seedlings in darkness to that in light. D1 stood for the *zmga3ox* seedlings treated by GA_3_ at 0 to 24 h; D2 stood for the *zmga3ox* seedlings treated by GA_3_ at 25 to 48 h. Data were collected for two diurnal cycles and were presented as mean  ±  SD (n  =  3). Different letters indicate significant differences between the WT, *zmga3ox* and *zmga3ox* + GA_3_ plants calculated by Fisher’s LSD (*p* < 0.05). (**d**) The phenotype (Bar = 1.0 cm) and cell morphology (Bar = 50 μm) of *zmga3ox*, *zmga3ox* + GA_3_ and WT seedlings. (**e**) The number of leaf cells of *zmga3ox*, *zmga3ox +* GA_3_ and WT seedlings in light and darkness. Data were presented as mean  ±  SD (n  =  30). (**f**) The leaf cell length of the *zmga3ox*, *zmga3ox* + GA_3_ and WT seedlings in light and darkness. Different letters indicate significant difference between the WT, *zmga3ox* and *zmga3ox* + GA_3_ seedlings calculated by Fisher’s LSD (*p* < 0.05). Values were the means ± SD (n  =  200).

The leaf length of *zmga3ox* was lower than that of wild-type (WT), with the addition of exogenous GA_3,_ the leaf length of *zmga3ox* + GA_3_ gradually increased and reached a length between that of the zmga3ox and the WT (Figure 1a). Similarly, the leaf elongation rate was higher in WT than *zmga3ox*, and the leaf elongation rate in light was higher than that in darkness (Figure 1b). The GA_3_ treatment could increase the leaf elongation rate in *zmga3ox*, while *zmga3ox* + GA_3_ had a higher leaf elongation rate in darkness than in light. Moreover, the V_D_/V_L_ value of WT was higher than *zmga3ox* in the D1 and D2 processes, but *zmga3ox* + GA_3_ had a higher V_D_/V_L_ value compared with WT in both processes (Figure 1c). In addition, the cell growth rate in WT and *zmga3ox* both presented lower in darkness than in light, while WT had a higher cell growth rate compared to *zmga3ox*, and GA_3_ treatment could increase the cell growth rate in *zmga3ox* (Figure 1d,e). Importantly, there was no significant difference in the cell length of WT between the reproduction in light and darkness, but the cell length of *zmga3ox* from the reproduction in darkness was shorter than that in light (Figure 1f). GA_3_ treatment significantly promoted cell elongation in *zmga3ox*, while *zmga3ox* + GA_3_ had longer cell length from the proliferation in darkness than that in light.

### 2.2. Metabolic Characteristics in the GA-Modulated Rhythm Growth

**Figure 2:**
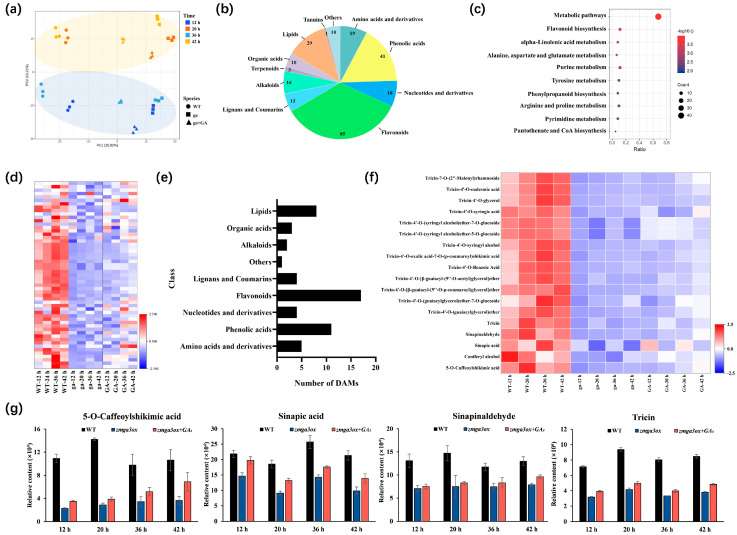
Metabolic profile and pathway analysis of GA-regulated metabolites. (**a**) Principal component analysis (PCA) of accumulation pattern showed two distinct groups: darkness (yellow) and light (blue) for samples taken at 4 time points in *zmga3ox*, *zmga3ox* + GA_3_ and WT. (**b**) The number and species of differentially accumulated metabolites (DAMs) in metabolome data. (**c**) KEGG enrichment analysis for all the DAMs regulated by GA_3_ in *zmga3ox*. The color of the point represents p, and the size of the point represents the number of DAMs. (**d**) Heat map of 55 DAMs upregulated by GA_3_ treatment. (**e**) The number and species of 55 DAMs upregulated by GA_3_ in metabolome data. The horizontal coordinate represents the number of DAMs, and the vertical coordinate represents the category. (**f**) Heat map of 18 DAMs related to lignin synthesis and regulated by GA_3_. (**g**) Relative content of metabolites involved in lignin synthesis and accumulated rhythmically, respectively, 5-O-Caffeoylshikimic acid, sinapic acid, sinapinaldehyde and tricin. Data were shown as the mean  ±  SD (n  =  3).

The time series metabolic profiles of WT, *zmga3ox*, and *zmga3ox* + GA_3_ seedling were tested through untargeted metabolomics (Figure S1a), and principal component analysis (PCA) and cluster analysis of metabolome data set showed that different treatments were well distributed according to light (12 h, 36 h) and darkness (20 h, 42 h) time points (Figure 2a and Figure S1b). The differentially accumulated metabolites (DAMs) involved 11 species were detected (Figure 2b and Figure S1c–e), of which flavonoids (34.7%) were the most, followed by phenolic acids (16.7%) and lipids (11.8%) (Figure 2b). KEGG annotation for all the DAMs indicates the main enrich in “Metabolic pathways”, “Flavonoid biosynthesis”, “Phenylpropanoid biosynthesis”, and “Alanine, aspartate and glutamate metabolism” (Figure 2c).

Of the DAMs, 55 DAMs were lower in *zmga3ox* than WT while upregulated by GA_3_ treatment (Figure 2d, Table S1). Among them, there were 17 (30.9%) flavonoids, 11 (20%) phenolic acids (Figure 2e). Among the 17 flavonoid metabolites, 14 (82.4%) DAMs were tricin and its derivatives. Among the 11 phenolic acids, 5-O-caffeoylshikimic acid, coniferyl alcohol, sinapic acid and sinapinaldehyde were observed. In addition, 12 metabolites (21.8%) accumulated with circadian rhythm characteristics and were upregulated by GA_3_ treatment in *zmga3ox*, including 5-O-caffeoylshikimic acid, sinapic acid, sinapinaldehyde, tricin, etc. (Table S1); 5-O-caffeoylshikimic acid, sinapinaldehyde, tricin were accumulated more in darkness than in light, and the opposite occurred in sinapic acid (Figure 2f,g). Meanwhile, 5-O-caffeoylshikimic acid, sinapic acid, sinapinaldehyde and tricin were upregulated by GA_3_ treatment in *zmga3ox* (Figure 2f) Inversely, 71 DAMs were higher in *zmga3ox* than WT while downregulated by GA_3_ treatment (Figure S1f, Table S1). There were 32 (45%) flavonoids and 14 (19.7%) phenolic acids (Figure S1g). Of these 71 metabolites, 10 (14.1%) showed a circadian accumulation and downregulated by GA_3_ treatment in *zmga3ox*, including salicylic acid, L-ornithine, xanthine, jasmonic acid, eriodictyol, etc. (Table S1).

### 2.3. Temporal Dynamics of the Transcriptional Response in GA-Modulated Rhythm Growth

**Figure 3:**
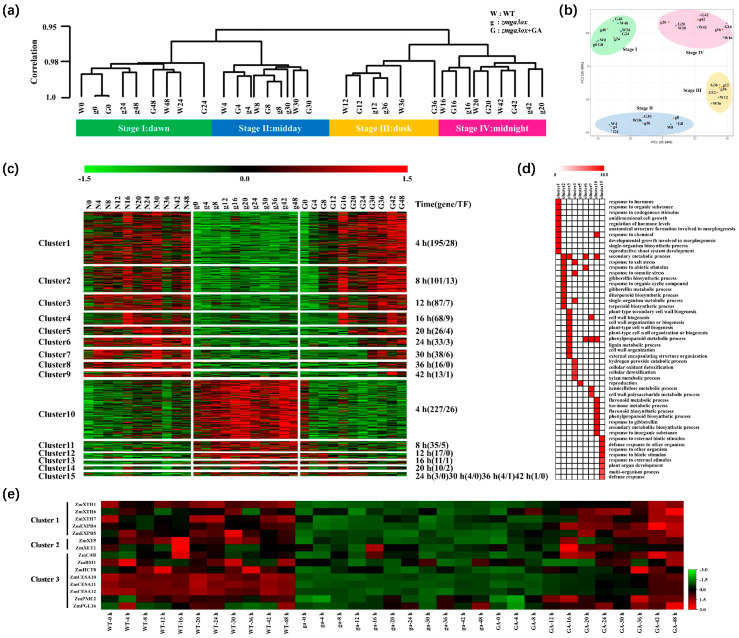
Analysis of temporal clustering and functional categories of GA-responsive genes. (**a**) Hierarchical clustering dendrogram showed two different data sets: Stages I and II (dawn and midday), stages III and IV (dusk and midnight). (**b**) PCA of the transcriptome data showed four categories for samples taken at 11 time points in *zmga3ox*, *zmga3ox* + GA_3_ and WT. Green for stage I: dawn, blue for stage II: middy, yellow for stage III: dusk, pink for stage IV: midnight. (**c**) Analysis of time series expression pattern of differentially expressed genes (DEGs) in *zmga3ox*, *zmga3ox* + GA_3_ and WT according to their sensitivity to GA_3_. The heatmap of the cluster analysis of DEGs was based on the log_2_ (fold-change) values of genes at 11 time points. (**d**) GO functional categories enriched of DEGs in different gene clusters, significant categories (Q < 0.05) were displayed. (**e**) Time series expression profile of cell wall synthesis and expansion genes in response to GA_3_. The heatmap of the cluster analysis was based on the log_2_ (fold-change) values of DEGs at 11 time points.

With paired-end Illumina sequencing technology, 15 billion clean reads were obtained; 92.4% reads could be mapped to the maize B73 reference genome (RefGen_V4), and there was a high correlation between biological replicates (average r^2^ = 0.949) (Figure S2a). PCA was performed on the transcriptome data set, and the different treatments were well compartmentalized according to the time points of light (4 to 12 h and 30 to 36 h) and darkness (0 h, 16 to 24 h, and 42 to 48 h) (Figure 3a). The cluster analysis showed that the cluster was divided into two periods of 0 to 24 h and 30 to 48 h (Figure 3b). At all time points, there were 1014 differentially expressed genes (DEGs) between WT and *zmga3ox*, while GA_3_ treatment could gradually decrease the number of DEGs between WT and *zmga3ox* + GA_3_ along with treatment time extension (Figure S2b,c).

By clustering the expression patterns, DEGs could be divided into 15 clusters (Figure 3c, Table S2). The clusters of C1 to C9 generally presented the upregulated expression patterns of DEGs between WT and *zmga3ox*, while their contrary expression patterns were observed in the clusters of C10 to C15. With the extension of GA_3_ treatment time, the DEG expression of *zmga3ox* + GA_3_ in C1 to C15 clusters gradually recovered to WT. Obviously, among the 577 (including 71 TFs) upregulated genes in GA_3_-treated *zmga3ox*, DEGs of C1 to C4 clusters responded to GA_3_ treatment faster, and the number of DEGs was higher. Correspondingly, 362 downregulated genes (including 35 TFs) in GA_3_-treated *zmga3ox* were gained in C10 to C15 clusters, while the number of DEGs was greatest in the C10 cluster and responded to GA_3_ treatment quickly.

The Gene Ontology (GO) annotation was performed, respectively, for gene function annotation and classification for C1 to C15 cluster (Figure 3d). The DEGs of the C1 cluster were mainly enriched in the ‘response to hormone’, ‘unidimensional cell growth’, ‘regulation of hormone levels’, ‘morphogenesis’ and ‘reproductive shoot system development’. The expression levels of DEGs such as *ZmGA2ox910*, *ZmIAA34/5/22*, and *ZmZIM28/30* in *zmga3ox* were lower than those in WT (Figure S2d), while they could be upregulated by GA_3_ treatment in *zmga3ox*. Meanwhile, the DEGs related to cell wall expansion, such as *ZmXTH1*, *ZmXTH6*, *ZmXTH7*, *ZmEXPB4* and *ZmEXPB5* (Figure 2e), were also upregulated by GA_3_ treatment in *zmga3ox*. The DEGs of the C2 cluster were mainly related to the ‘secondary metabolic process’, ‘stress response’, ‘GA biosynthetic and metabolic process’, and ‘terpenoid and diterpenoid biosynthetic process’, and the DEGs such as *ZmGA2ox2/3/6/12*, *ZmXT5*, *ZmXET1* and *Zm4CL* (Figure 2e and Figure S2d) were upregulated by GA_3_ treatment in *zmga3ox*. Moreover, the DEGs of C3 and C4 clusters were mainly enriched in the cell growth and cell wall synthesis, which included the genes involved the biosynthesis of the cellulose (*ZmCESA10*, *ZmCESA11* and *ZmCESA12*), lignin (*ZmC4H* and *ZmHCT8*), pectin (*ZmPGL16* and *ZmPME2*) (Figure 2e), and biosynthesis of intermediates such as various sugars. In addition, the DEGs of C5 to C7 clusters were mainly enriched in the ‘secondary metabolic process’, ‘response to abiotic stimulus’, and ‘cell wall biogenesis’. Furthermore, the DEGs of the C10 cluster were mainly enriched in ‘secondary metabolic process’, ‘hormone metabolic’, and ‘response to gibberellin’, while those of the C15 cluster were mainly enriched in the ‘defense response to other organism’, ‘plant organ development’, and ‘response to biotic stimulus’. The DEGs such as *ZmGA20ox1/4/5/6/9*, *ZmGID1/2*, *ZmLOX3/6*, and *ZmCKX6/10* were downregulated by GA_3_ treatment in *zmga3ox* (Figure S2d).

### 2.4. Expression Profile and Pathway Enrichment of Circadian Genes Involved in GA-Modulated Rhythm Growth

**Figure 4:**
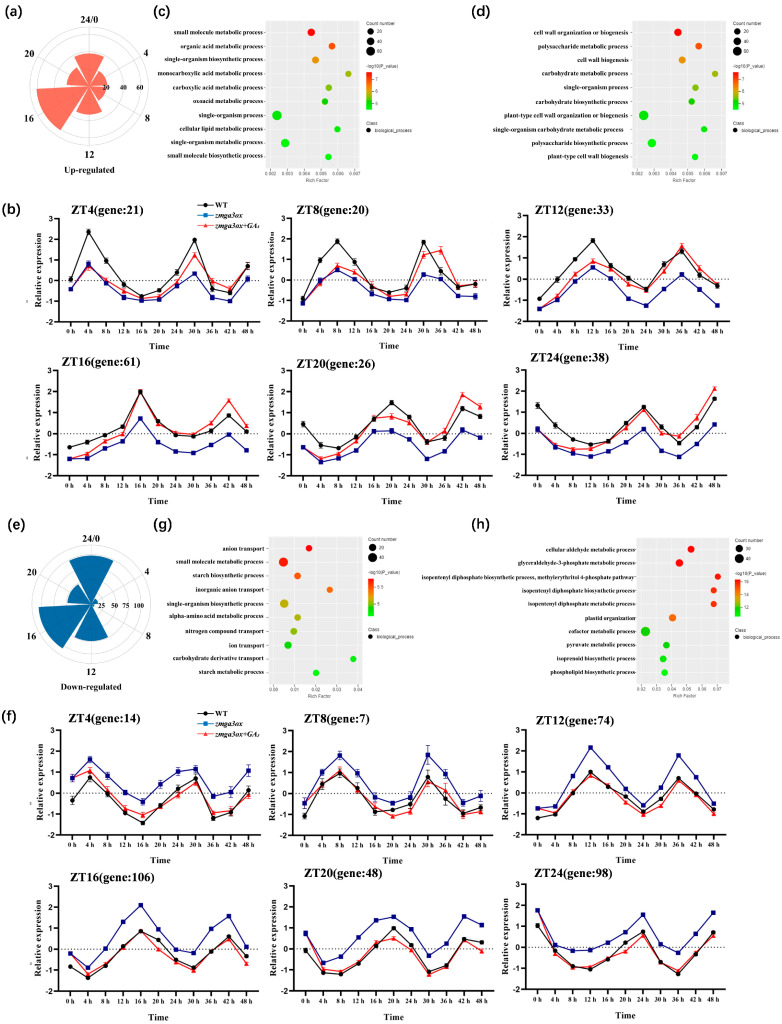
Analysis of circadian gene expression patterns and responsive pathway. (**a**) Statistics of the number of circadian genes with upregulated expression induced by GA_3_ at different time points. (**b**) The number and expression pattern of circadian genes upregulated by GA_3_ reached the peak expression, ZT4 expression peaked at 4 h after GA_3_ treatment, and ZT8 peaked at 8 h, ZT12, ZT16, ZT20, ZT24 and so on. (**c**) Pathway enrichment analysis of upregulated DEGs in light. The color of the point represents p, and the size of the point represents the number of DEGs. (**d**) Pathway enrichment analysis of upregulated DEGs in darkness. The color of the point represents p, and the size of the point represents the number of DEGs. (**e**) Statistics of the number of circadian genes with downregulated expression induced by GA_3_ at different time points. (**f**) The number and expression pattern of circadian genes downregulated by GA_3_ reached the peak expression, ZT4 expression peaked at 4 h after GA_3_ treatment, and ZT8 peaked at 8 h, ZT12, ZT16, ZT20, ZT24 and so on. (**g**) Pathway enrichment analysis of downregulated DEGs in light. The color of the point represents p, and the size of the point represents the number of DEGs in light. (**h**) Pathway enrichment analysis of downregulated DEGs in darkness. The color of the point represents p, and the size of the point represents the number of DEGs.

To explore the characteristics of the circadian rhythm of GAs modulated leaf growth, the 3702, 4101 and 2195 circadian genes were, respectively, identified in WT, *zmga3ox* and *zmga3ox* + GA_3_ (Figure S3a,b). A total of 1204 conservatively expressed circadian genes were identified (Figure S3c), and the reported classical circadian genes including *CCA1*, *LHY1*, *PRRTF1*, and *PRRH1* were observed (Figure S3d). After that, 546 circadian genes were DEGs regulated by GA_3_, while 199 DEGs were lower in *zmga3ox* than WT, which were upregulated by GA_3_ treatment in *zmga3ox* (Table S3). The number of upregulated DEGs involved circadian rhythm was higher in darkness than those in light (62.81%) (Figure 4a,b). Moreover, GA_3_ treatment upregulated the amplitude of the circadian gene expression spectrum, but did not move phase. The circadian genes with the peak expression in light (ZT4, ZT8, and ZT12) were mainly involved in metabolic processes, including ‘small molecule metabolism and biosynthetic’, ‘single-organism metabolism and biosynthetic’, ‘organic acid metabolic’, ‘oxoacid and carboxylic acid metabolic’, and ‘cellular lipid metabolic’ (Figure 4c). The circadian genes enriched in darkness (ZT16, ZT20, and ZT24) mainly acted on ‘cell wall organization or biogenesis’, ‘carbohydrate biosynthetic and metabolism’, and ‘polysaccharide biosynthetic and metabolism’ (Figure 4d).

A total of 347 circadian genes were upregulated in *zmga3ox* and could also be restored to WT expression level after GA_3_ treatment (Table S3). Meanwhile, the number of circadian genes regulated by GA_3_ treatment was higher in darkness (72.6%) than those in light (Figure 4e,f). These DEGs were related to ‘ion transport’, ‘starch metabolic and biosynthetic’, ‘small molecule and alpha-amino acid metabolic’ and ‘nitrogen compound and carbohydrate derivative metabolic’ processes in light (ZT4, ZT8, and ZT12) (Figure 4g). In darkness (ZT16, ZT20, and ZT24), the downregulated circadian genes by GA_3_ in *zmga3ox* were mainly involved in ‘aldehyde metabolic’, ‘isopentenyl diphosphate biosynthetic and metabolic’, ‘cofactor metabolic’, ‘isoprenoid and phospholipid biosynthetic’ and ‘pyruvate metabolic’ processes (Figure 4h).

### 2.5. GAs-Regulated the Circadian Genes and Rhythmically Accumulated Metabolites Were Enriched in the Lignin Synthesis Pathway

**Figure 5:**
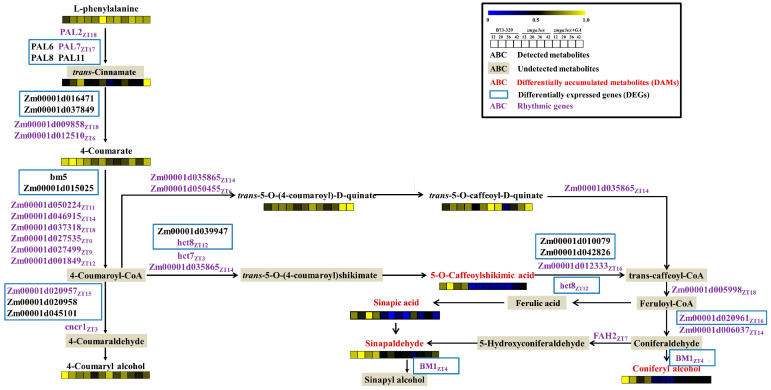
Comprehensive analysis of DEGs and DAMs in the pathway of lignin synthesis.

The DAMs and DEGs involved in the lignin synthesis pathway were then integrated and analyzed comprehensively (Figure 5). Among the lignin synthesis pathways, 17 DEGs were regulated by GA_3_, and the circadian genes including *ZmBM1*, *ZmHCT8*, *ZmPAL17*, *Zm00001d020957* and *Zm00001d020961*. Four lignin monomers or intermediate metabolites were upregulated by GA_3_ in *zmga3ox*, including coniferyl alcohol, sinapic acid, sinapinaldehyde and 5-O-caffeoylshikimic acid. The accumulation of sinapic acid, sinapinaldehyde and 5-O-caffeoylshikimic was not only regulated by GA_3_ treatment in *zmga3ox*, but also presented circadian accumulation (Figure 2g). The metabolites accumulation of sinapinaldehyde and 5-O-caffeoylshikimic acid in light was lower than that in darkness, while sinapic acid was the opposite. The conversion of 5-O-caffeoylshikimic acid was catalyzed by *ZmHCT8*, and the conversion process of sinapyl alcohol synthesized from sinapinaldehyde was catalyzed by *ZmBM1*.

### 2.6. Transcriptional Regulatory Network of GA-Regulated Lignin Biosynthesis

**Figure 6:**
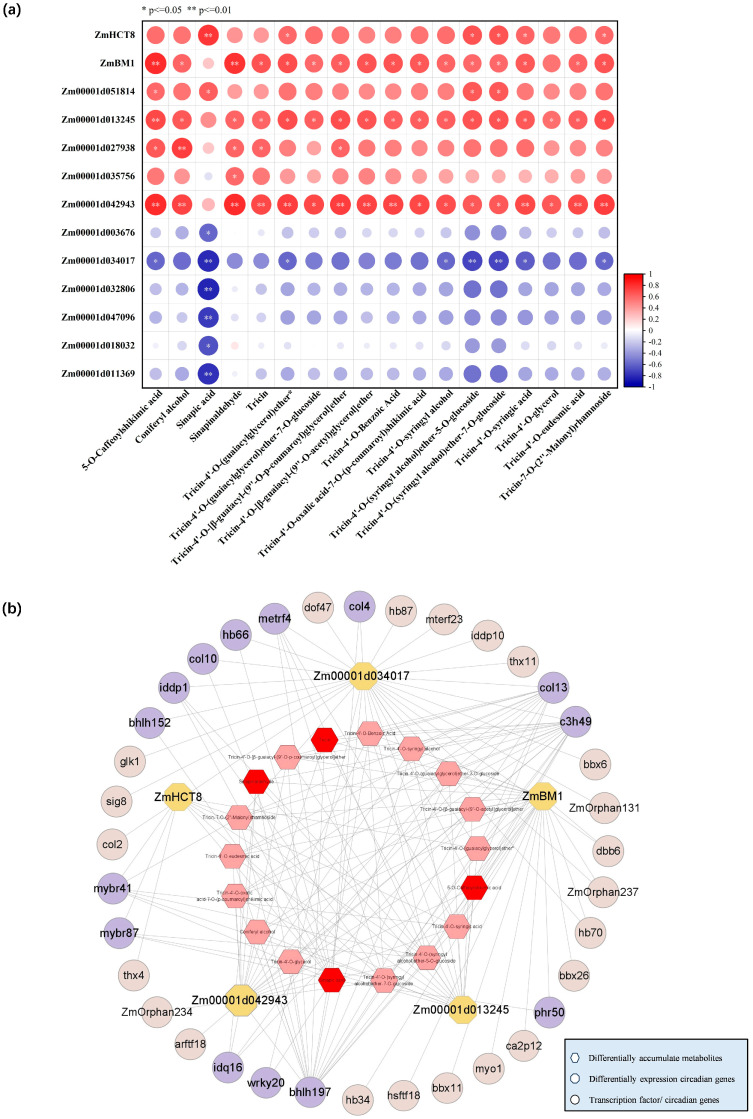
Association analysis of DEGs and DAMs in lignin synthesis pathways. (**a**) Correlation analysis of circadian DEGs and DAMs in lignin synthesis pathway. Red represents a positive correlation, blue represents a negative correlation, **: *p* < 0.01; *: *p* < 0.05. (**b**) Network diagram of DEGs and DAMs. Hexagon represents the DAMs in lignin synthesis, pink is the DAMs, red is the rhythmically accumulated DAMs, octagon represents the target genes related to cell wall synthesis, and circle represents the transcription factors co-expressed with the target genes, in which purple is associated with the accumulation of lignin synthesis metabolites and gray is not associated.

The correlation between the expression of circadian DEGs related to cell wall synthesis and lignin synthesis DAMs was analyzed, and 13 genes were identified (Figure 6a), among which lignin synthase genes *ZmBM1* and *ZmHCT8*, *Zm00001d042943* (UXS1), *Zm00001d013245* (UGD2) and *Zm00001d034017* (Exhydrolase II) were significantly correlated with DAMs. Then, the gene regulatory network analysis (GRN) was performed with this five circadian DEGs. As shown in Figure 6b and Figure S5a, a total of 81 transcription factors were identified (Table S4), and 35 rhythmical transcription factors including *ZmARF18*, *ZmDOF47*, *ZmMYBr41/87*, *ZmHB34/70*, and *ZmbHLH197* were obtained. Between the 35 rhythmical transcription factors, 14 were correlated with DAMs in lignin synthesis (Figure 6b and Figure S4), including *ZmMYBr41*, *ZmMYBr87*, *ZmbHLH197*, *ZmWRKY20*, *ZmC3H49*, and *ZmHB66*. Among the 18 DAMs associated with lignin synthesis, they were all associated with different cell wall synthesis or expansion genes, among which sinapinaldehyde, sinapic acid, 5-O-caffeoylshikimic acid, and two tricin derivatives had a significant correlation with cell-wall-related DAMs (Figure 6b), Most importantly, sinapinaldehyde, sinapic acid, 5-O-caffeoylshikimic acid and tricin were DAMs that were accumulated rhythmically.

### 2.7. Identification of Key Transcriptional Regulators of GA-Regulated Lignin Biosynthesis

**Figure 7:**
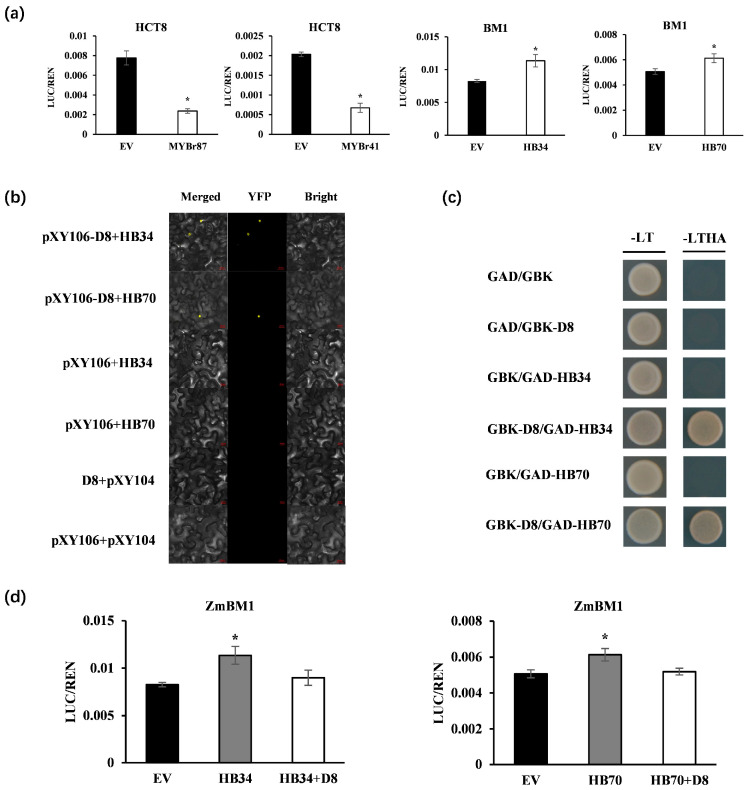
Transcriptional regulation of target genes for cell wall synthesis by candidate transcription factors and verification of interactions between candidate transcription factors and *ZmD8*. (**a**) Validation of the binding and regulation of transcription factors to the cis-elements in corresponding target genes by dual-luciferase assays. Data are presented as mean  ±  SD (n  =  3), * indicates significant difference. Student’s *t*-test was used in significance statistical analysis (*p* < 0.05). (**b**) Interaction between *ZmHB34/70* and *ZmD8* by BiFC assay. The construct combinations were cotransfected in *N. benthamiana* leaves. The yellow fluorescent protein (YFP) signal was detected by confocal microcopy after 48 h of incubation. Scale bars, 20 μm. (**c**) Yeast-two-hybrid assays demonstrated the interaction of *ZmHB34/70* with *ZmD8*. The transformants were screened on SD/-Trp-Leu and SD/-Trp-Leu-His-Ade medium. (**d**) Validation of the binding and regulation of *ZmD8* co-transformation with *ZmHB34/70* on the transcriptional activity of *ZmBM1* by dual-luciferase assays. Data are presented as mean  ±  SD (n  =  3), * indicates significant difference. Student’s *t*-test was used in significance statistical analysis (*p* < 0.05).

To analyze the regulatory network of lignin synthesis regulated by GAs and verify the reliability of the transcriptional regulatory network, the binding sites of transcription factors and target genes were analyzed (Figure S5), and a dual-luciferase assay was performed on the interaction of transcription factors and target genes (Figure 7a). The expression of *ZmHCT8* was repressed by *ZmMYBr41* and *ZmMYBr87*. *ZmHB34* and *ZmHB70* promoted the transcription expression of *ZmBM1*.

The role of GAs signaling in the regulation of lignin synthase genes by candidate transcription factors was then explored. DELLAs, a negative regulatory element of GA signaling, was taken as the key regulatory factor. To verify whether DELLAs were the key element in the signaling pathway in which GA signal and circadian rhythm signal co-regulate lignin synthesis, BiFC and Y2H were used to verify DELLAs interaction with *ZmHB34/70* (Figure 7b,c). *ZmD8* could physically bind to *ZmHB34/70* in vitro. Moreover, compared with *ZmHB34/70*, the promoting effect of ZmD8 and *ZmHB34/70* co-conversion on *ZmBM1* was weakened (Figure 7d). Therefore, *ZmHB34/70* might be a potential regulator of the GA-regulated lignin synthesis pathway.

## 3. Discussion

GA signals regulate plant growth and development, and many studies have shown that the manipulation of GA synthesis genes or signal response genes can modulate cell proliferation or elongation [42,43]. Here, exogenous GA_3_ treatment promoted the cell elongation for leaf growth in *zmga3ox*, resulting in a length between that of the *zmga3ox* and the WT. (Figure 1). This was consistent with the results of previous studies that GAs promote the longitudinal growth of maize leaf elongation [44,45]. Interestingly, the leaf elongation rate and cell growth presented higher in WT and *zmga3ox* seedlings in light than those in darkness, while the cell length of *zmga3ox* propagated in darkness was shorter than that in light. It has been proved that DELLA proteins were regulatory components of the interaction between GA signal and circadian signal [41], resulting in significant growth differences in plants treated with GAs at night [15]. In this study, *zmga3ox* + GA_3_ had a higher leaf elongation rate and a longer cell length in darkness than that in light. These results indicated the maize leaf growth presented a circadian rhythm, and GAs could modulate the rhythmic growth without changing the circadian rhythm.

By analyzing the time point and function of the gene response to GAs (Figure 3), the genes involved in cell wall synthesis and hormone signaling were the earliest to respond to GA_3_ treatment in *zmga3ox*. Meanwhile, the *ZmGA20ox1/4/5/6/9* were downregulated at 4 h after GA_3_ treatment in *zmga3ox*, whereas the *ZmGA2ox2/3/6/9/10/12/13* were upregulated at 4 h and 8 h after GA_3_ treatment. Similarly, the *ZmGA20ox2* is rapidly downregulated 15 min after GA treatment [46], and GAs regulate the expression of cell wall organization and modification genes [47,48,49,50,51,52,53,54]. Here, the DEGs of the cell wall expansion such as *EXPs* (*ZmEXPB4* and *ZmEXPB5*) and *XTHs* (*ZmXTH1*, *ZmXTH6*, and *ZmXTH7*) were upregulated 4 h after GA_3_ treatment in *zmga3ox* seedlings, and then the DEGs of the cell wall synthase genes (*ZmCESA10/11/12*, *ZmC4H*, *ZmHCT8*, *ZmPGL16*, and *ZmPME2*) were upregulated at 8 h and 12 h. These suggested that the expression of cell wall organization and modification genes presented the time response series after GA_3_ treatment in *zmga3ox* seedlings, which might be involved in GAs-mediated leaf rhythmic growth.

Most circadian gene expression presented similar patterns between WT and *zmga3ox* seedlings (Figure 4), but that of *zmga3ox* showed a difference in expression levels compared to WT, while the expression levels of those in GA_3_-treated *zmga3ox* seedlings could be restored to similar levels of those in WT. As is known, circadian genes are highly expressed at distinct times during light and darkness, and the peak of circadian gene expression can be relatively representative of the time at which genes may be active [23,24,25]. In this study, the number of circadian DEGs was higher in darkness than in light after GA_3_ treatment in *zmga3ox* seedlings, and the circadian DEGs enriched in cell wall synthetic pathways mostly presented the peak in darkness, which suggested that GAs could have a greater effect on cell wall synthesis in darkness. Several researchers have identified some cell wall synthase genes (*CESA4/6* and *CAD4/6*) as circadian genes [13,14]. Here, the cell wall organization and modification genes, such as *ZmBM1*_ZT4_, *ZmHCT8*_ZT12_, *ZmCESA8*_ZT16_, *ZmEXPA4*_ZT20_, *ZmXTH3*_ZT24_, *Zm00001d027938*_ZT16_ (GUX2), *Zm00001d042943*_ZT20_ (UXS1), and *Zm0000013245*_ZT16_ (UGD2), were identified as the circadian genes, which could be upregulated by GA_3_ treatment in *zmga3ox* seedlings. All of these genes were associated with cell wall synthesis [55,56,57,58,59,60]. These results indicated that GAs could affect the circadian genes related to the cell wall synthesis process to promote the leaf rhythmic growth of *zmga3ox* seedlings.

GAs may modulate cell wall relaxation and biosynthesis process to control the cell shape [7,8,9]. The lignin is a phenolic polymer among plant secondary metabolites, while the lignin and flavonoid synthesis belongs to phenylpropane synthesis [61,62,63]. In this study, GA_3_ treatment mainly regulated the metabolites of secondary metabolic pathways such as flavonoids and phenolic acids in *zmga3ox* seedlings (Figure 2). Among DAMs of the flavonoids upregulated by GA_3_ treatment, 14 (82.3%) were tricin and its derivatives. Many studies have shown that tricin as a monomer of lignin exists in lignin components from various monocotyledonous plants [64,65,66,67,68,69,70]. Furthermore, DAMs of the flavonoids downregulated by GA_3_ are mainly enriched in phenylpropane synthesis pathway including flavonoids and flavonol metabolites [62,63]. Among DAMs of the phenolic upregulated by GA_3_ treatment, four (36.3%) were lignin synthesis metabolites, including 5-O-caffeoylshikimic acid, sinapinaldehyde, sinapic and coniferyl alcohol. The coniferyl alcohol and sinapyl alcohol are the monomers of lignin [71,72]. In addition, DAMs of the phenolic acids downregulated by GA_3_ are mainly enriched in the phenylpropane synthesis pathway. This suggests that GAs could modulate the accumulation of metabolites in flavonoids and the phenylpropane synthesis pathway enriched in lignin synthesis during the GA-mediated leaf growth process.

It was very interesting that 22 DAMs induced by GA_3_ treatment showed rhythmic accumulation characteristics in *zmga3ox* seedlings, and 4 of them were the metabolites of the lignin synthesis pathway, respectively 5-O-caffeoylshikimic acid, sinapinaldehyde, sinapic acid and tricin (Figure 2g). Moreover, the accumulation of 5-O-caffeoylshikimic acid, sinapinaldehyde and tricin was higher in darkness than in light, while the accumulation of sinapic acid showed an opposite pattern. The 5-O-caffeoylshikimic acid can be catalyzed by *p*-coumaroyl-CoA (C3H) and hydroxycinnamoyl-CoA shikimate/quinate hydroxycinnamoyl tranferase (HCT) to caffeoyl coenzyme A [73,74,75,76]. Importantly, *ZmHCT8* encoding HCT was identified as a circadian DEG here. After that, sinapinaldehyde is produced by cinnamyl-alcohol dehydrogenase (CAD) in the formation of sinapyl alcohol, as one of the lignin monomers [77,78,79,80,81,82]. The *ZmBM1* encoding the CAD was identified as circadian DEGs in this study. Moreover, the formation of the secondary cell wall indicates the cell stops growing, and the lignin is an important component of the secondary wall [7]. Therefore, GAs could upregulate the expression of *ZmBM1* and *ZmHCT8* to modulate the lignin biosynthesis for controlling leaf growth.

**Figure 8:**
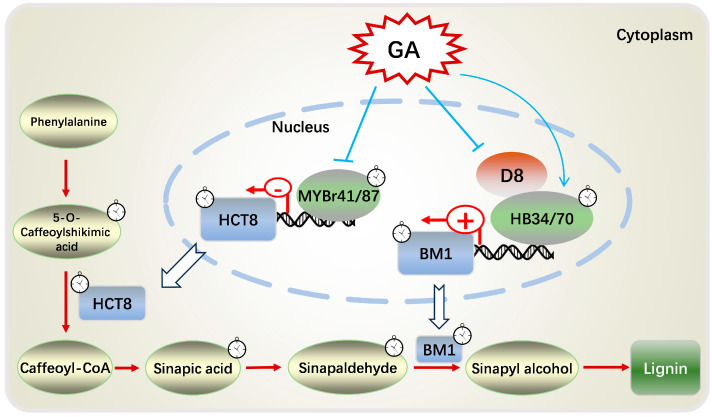
Proposed working model of GA-mediated regulation of circadian rhythm metabolism of lignin. GAs promoted the rhythmic expression of *ZmHCT8* and *ZmBM1* by regulating the transcriptional expression of circadian DEGs *ZmMYBr41/87* and *ZmHB34/70*. Additionally, GAs interfered with the inhibitory effect of *ZmHB34/70* on *ZmBM1* through the interaction module of *ZmD8* and *ZmHB34/70*, then affected rhythmic accumulation of 5-O-caffeoylshikimic acid, sinapic acid and sinapaldehyde, and influenced the lignin synthesis.

The correlation analysis obtained five circadian DEGs associated with cell wall synthesis including *ZmBM1*, *ZmHCT8*, *Zm00001d042943* (UXS1), *Zm00001d013245* (UGD2), and *Zm00001d034017* (Exhydrolase II), which were significantly correlated with 18 lignin synthesis metabolites and regulated by GA_3_ treatment in *zmga3ox* seedlings (Figure 6a). The *Zm00001d034017* homologous gene *At5g20940* encodes a beta-glucosidase involved in xyloglucan metabolism, while the xyloglucan is a hemicellulose polysaccharide present in the cell wall [83]. GRN analysis of the above five cell-wall-associated circadian genes identified 35 rhythmically expressed transcription factors, including *ZmARF18*, *ZmHB34*, *ZmHB70*, *ZmbHLH197*, *ZmDOF47*, *ZmMYBr41* and *ZmMYBr87*, and then 14 of those were correlated with 18 DAMs in lignin synthesis, including *ZmMYBr41* and *ZmMYBr87* and *ZmbHLH197*. These indicated that the rhythmically expressed transcription factors including *ZmARF18*, *ZmHB34*, *ZmHB70*, *ZmbHLH197*, *ZmDOF47*, *ZmMYBr41* and *ZmMYBr87* could participate in regulating the expression of the functional genes involved in cell wall synthesis. Then, *ZmMYBr41* and *ZmMYBr87* were the most correlated transcription factors among the genes co-expressed with *ZmHCT8*, and the expression of *ZmHCT8* was repressed by *ZmMYBr41* and *ZmMYBr87*. *ZmMYBr41* and *ZmMYBr87* were negatively correlated with lignin synthesis metabolites, and downregulated by GA_3_ in *zmga3ox*. This suggested that GAs could inhibit the expression of *ZmMYBr41* and *ZmMYBr87* to activate the expression of *ZmHCT8* for modulating the accumulation of 5-O-caffeoylshikimic acid in lignin biosynthesis (Figure 8). *ZmMYBr41* turns out to be a gene associated with plant height [84], and *ZmMYBr87* is related to cell wall synthesis [85], which further proves the involvement of *ZmMYBr41* and *ZmMYBr87* in regulating cell wall synthesis during leaf cell growth.

The *ZmHB34*, *ZmHB70* and *ZmBM1* had the same expression pattern, while were upregulated by GA_3_ in *zmga3ox*. Meanwhile, the *ZmHB34* and *ZmHB70* promoted the expression of *ZmBM1*, which suggested that *ZmHB34* and *ZmHB70* were the positive regulators of the lignin synthesis. Many studies showed that GAs may regulate the expression of cell-wall-related genes through DELLA interaction with transcription factors [86,87,88,89,90]. Here, *ZmD8* might interact with *ZmHB34* and *ZmHB70* to inhibit the expression of *ZmBM1*, affecting the conversion of sinapinaldehyde to sinapyl alcohol in lignin synthesis (Figure 8). Consequently, the proposed working model was built that GAs could regulate the expression of the rhythmic transcription factors *ZmMYBr41/87* and *ZmHB34/70* to modulate the lignin biosynthetic genes *ZmHCT8* and *ZmBM1* for manipulating the leaf elongation growth (Figure 8). In addition, GAs-mediated leaf rhythmic growth was involved in regulating the cell number and length, and it would be further study to explore how GAs modulated cell proliferation for responding to the leaf rhythmic growth.

## 4. Materials and Methods

### 4.1. Plant Materials, Growth Conditions, and Material Collection

The *zmga3ox* (GRMZM2G036340) mutant was obtained from the Maize Functional Genomic Project of China Agricultural University. The *zmga3ox* knocked out the 34bp fragment of the ZmGA3ox gene in the maize inbred line ND101 via CRISPR/Cas9 [91]. Seeds of ND101 (wild-type) and *zmga3ox* were sterilized in a 10% (*v*/*v*) H_2_O_2_ solution for 20 min and washed 5 times with distilled water. Then, seeds were germinated in the sand in a growth chamber, at 28/22 °C with a 16/8 h light/dark cycle and 70%−80% relative humidity. After 7 days, seedlings with two visible leaves were transferred to nutrient solution for 4 days, and the nutrient solution was replaced every 2 days. The modified Hoagland solution contains 0.5 mM MgSO_4_, 0.1 mM KH_2_PO_4_, 1 mM CaCl_2_, 0.1 mM EDTA-Fe, 2 mM KNO_3_, and micronutrients (0.03 mM H_3_BO_3_, 0.0025 mM ZnSO_4_, 0.008 mM CuSO_4_, 0.005 mM MnSO_4_, and 0.0003 mM (NH_4_)_6_Mo_7_O_24_, and pH 5.8 [92]. Cultured in nutrient solution for 4 days, *zmga3ox* mutant plants were treated with 1 µM GA_3_, while WT and *zmga3ox* were treated with equal volume ethanol [91]. Transcriptome sampling was conducted from 0 h to 24 h after GA_3_ treatment at intervals of 4 h and 24–48 h at intervals of 6 h. Two biological replicates were taken at each time point, and the leaves of three seedlings were taken as one replicate. We obtained a total of 64 samples of leaf for RNA-seq: 22 WT samples, 22 *zmga3ox* samples and 20 *zmga3ox* + GA_3_ samples. The difference was that only four time points were set for metabolomic sampling, namely two time points during the light (12 h, 36 h) and two time points in darkness (20 h, 42 h). Three biological replicates were taken at each time point, and the leaves of three seedlings were taken as one replicate.

### 4.2. Leaf Morphological Traits Measurements

Leaf cell morphology was observed using a TM4000 Scanning Electron Microscope scanning electron microscope (Hitachi, Tokyo, Japan). Fresh leaves were taken from positions in the mature area after 4 days of GA_3_ treatment and placed directly for observation after freezing in a scanning electron microscope observation room. The microscope viewing parameters were set to 100× and 15 kV. When the seedlings had grown the third visible leaf, GA_3_ was added at the beginning of light, and the length of the third visible leaf was measured at the same time. The measurement of leaf length was based on the distance between the junction of the root and stem and the leaf tip. The length of the third visible leaf was measured once more at the beginning of darkness, and the length of the maturation zone was represented by the added length of the leaf blade. This measurement was repeated for two consecutive light–dark cycles. The mature zone at the time of the GA3-treated light was determined by subtracting the first measured leaf length from the final leaf length and then clipping the growth length from the first light.

### 4.3. RNA Isolation, Transcriptome Sequencing, and Differential Gene Expression Analysis

The total RNA of all samples was isolated by the sTRIzol method (Invitrogen, Waltham, MA, USA). The quality of the purified RNA was evaluated with a NanoDrop 2000 (Thermo Fisher, Waltham, MA, USA) and Agilent 2100 (Agilent, Santa Clara, CA, USA). RNA-seq libraries were prepared according to the manufacturer’s protocol of the Illumina Standard mRNA-seq library preparation kit (Illumina, San Diego, CA, USA) and were sequenced to generate 150-nucleotide paired-end reads on a Nova platform (Illumina).

The B73 reference genome (RefGen_v4) [93] was downloaded from http://ensembl.gramene.org/Zea_mays/Info/Index, accessed on 1 January 2020. After removing low-quality reads using the (V2.5) software [94], Illumina sequencing reads were mapped to the B73 reference genome using Hisat2-2.0.4 [95] with default settings for parameters. The .bam files of uniquely mapped reads were used as inputs for the (V2.2.0) software [96], and FPKM values were calculated to measure the expression levels of genes. We calculated the Pearson correlation coefficient between biological replicates with the normalized expression levels of log_2_ (FPKM value +1). PCA was performed using the prcomp function in R software (R Team, 2013, V4.3.1) with default settings to facilitate graphical interpretation of relatedness among 11 different time points samples. The transformed and normalized gene expression values with log2 (FPKM +1) were used for hierarchical clustering, and the z-scores of the genes were used for the analysis of PCA.

Cuffdiff software in the Cufflinks (V2.2.0 version) software package [96,97] was used to calculate the differential expression level and significance of genes. Genes were considered as DEGs if they had a minimum 2-fold difference (| log2 (fold-change) | ≥ 1 and Q-value < 0.05) in expression at least one of the time points, as determined by Cuffdiff (V2.2.0 version). The heatmap of the expression patterns of each gene cluster was generated by MeV (V4.9 version) software. Each differential type of DEGs was evaluated for functional category enrichment using the function annotation module in AgriGO (v2.0) [98].

### 4.4. Identification and Analysis of Circadian Genes

Circadian genes oscillated for 24 h in transcriptome data were identified by both COSOPT and JTK_CYCLE with high confidence thresholds (Molecular timetable, CV ≥ 0.3 and R ≥ 0.9; JTK_CYCLE, BH.Q < 0.01) [99,100]. The FPKM value was used to fit the expression profile of each gene according to the cosine curve, CV (coefficient of variation) was used as a proxy for relative amplitude, and genes with CV ≥ 0.3 and R ≥ 0.9 were selected as circadian genes. Circadian genes with the same circular phase were divided into 1 group, with a total of 24 groups. The JTK_CYCLE algorithm was available as a computationally efficient R script. JTK_CYCLE accurately estimated the period, phase, and amplitude of cycling transcripts. BH.Q < 0.01 was used for multiple testing to consider circadian genes.

### 4.5. Metabolites Measurements, Data Processing and Metabolite Mining

The metabolites from freeze-dried samples were extracted overnight in a 70% methanol solution. The sample extracts were analyzed using a UPLC-ESI-MS/MS system (UPLC, SHIMADZU NexeraX2, Kyoto, Japan; MS, Applied Biosystems 4500 Q TRAP), Waltham, MA, USA. The MS data were processed using Analyst 1.6.3 software to obtain the total ion flow current and MRM detection of multimodal maps of mixed mass control samples. Based on the self-built metware database (MWDB), material characterization was carried out according to the information of the secondary spectrum. The signal intensity (CPS) of the characteristic ions was obtained in the detector by screening each substance with a triple quadrupole. The MultiaQuant software (V2.0) was used to process the MS data, integrate and correct chromatographic peaks, and export the integration data of the chromatographic peak area for preservation.

The raw data signals were processed using the Analyst 1.6.3 software (AB Sciex, Framingham, MA, USA). The original abundance of metabolites was log-transformed to normalize the data and for homogeneity of variance. PCA, cluster analysis, and orthogonal projections to latent structure-discriminant analysis (OPLS-DA) were carried out using R (http://www.r-project.org/) in accordance with previously described methods. Variable importance in projection (VIP) values of all metabolites from the OPLS-DA were extracted using the first component. The metabolites satisfying the following two criteria were selected as differential metabolites: (i) high confidence (VIP ≥ 1) in pairwise comparisons; (ii) a minimum of a 2-fold change or a maximum of 0.5-fold change (fold change ≥ 2 and fold change ≤ 0.5). The enrichment pathways of metabolites were analyzed based on the KEGG database with a *p*-value ≤ 0.05 established as the false discovery rate (FDR) for multiple tests, and metabolic pathway networks were constructed using Cytoscape (V3.8.2 version).

### 4.6. Dual-Luciferase Assays

The full-length coding region of ZmTFs was cloned into the vector pGreenII62-SK, these were all referred to as effectors. Empty pGreenII 62-SK vector was used as a control. The promoter sequence was selected from 2000bp upstream of the coding region and fused to the pGreenII0800 vector, and was used as the reporter. The effector and reporter were transferred into maize protoplasts by the PEG transformation method, and the Firefly luciferase (LUC) and Renilla luciferase (REN) activity was detected after 12 h culture by a dual-luciferase reporter assay system (Promega, Madison, WI, USA). The analysis was carried out using a Glomax Navigator (promega) according to the manufacturer’s instructions. Three independent experiments (biological replicates) were performed, and the Firefly luciferase (LUC) and Renilla luciferase (REN) activity were measured.

### 4.7. Gene Regulatory Network Analysis

The context likelihood of relatedness (CLR) algorithm method was used to identify highly correlated gene pairs of TFs. To construct the TF-related GRN, using CLR calculated correlation strength, comparing the mutual information (MI) between a TF and its gene pairs to the MI for all TFs and gene pairs in the background, which was calculated according to the expression similarity between the expression levels of TF-related gene. The formula was as follows: *f* (*Z*_*i*_, *Z*_*j*_) = SQRT (*Z*_*i*_^2^ + *Z*_*j*_^2^), where *Z_i_* is the *z* score between gene *i* and its background genes, and *Z_j_* is the *z* score between gene *j* and its background genes. According to the report published by Faith et al. [99], an f value [101] of more than 4.5 was identified as a highly correlated regulatory relationship, and the considering genes were nodes in the network. Cytoscape (v3.9.0) (ref. [86]) was used to visualize the network, generating an image using cytoscape with default parameters.

### 4.8. Yeast Two-Hybrid Assay

*ZmD8* was fused with the pGBKT7 vector. *ZmMYBr41/87* and *ZmHB34/54* were fused with the pGADT7 vector and co-transformed into the yeast strain Y2H Gold strain using the PEG/LiAc method. Growth on an SD-A-L-H-T medium was measured to verify interaction.

### 4.9. Bimolecular Fluorescence Complementation Assay

The coding region of *ZmD8* and *ZmMYBr41/87*, and *ZmHB34/70* were cloned into the linearized pXY106 and pXY104 vectors. The fusion vector was transferred to Agrobacterium strain GV3101, and the mixed bacterial solution was co-expressed in *N. benthamiana* leaves. After 48 h, the fluorescence of YFP in leaves was observed using confocal laser scanning microscopy (Leica TCS SP5; Leica Camera AG, Wetzlar, Germany).

### 4.10. Statistical Analysis

The data were statistically analyzed using SPSS statistics 26.0 (SPSS Inc., Chicago, IL, USA). One-way ANOVA with LSD and Duncan’s multiple range test (*p* < 0.05) was used. The histogram was generated with GraphPad Prism 8 (GraphPad Software Inc., San Diego, CA, USA, 2020).

## 5. Conclusions

The maize leaf presented a circadian rhythm elongation, and the rate of leaf elongation and cell growth was lower in darkness than in light. The *zmga3ox* seedling had a lower rate of leaf elongation and cell growth than WT, and GA_3_ treatment could restore the leaf growth of *zmga3ox*. The DEGs between WT and *zmga3ox* were mainly enriched in pathways related to hormone signaling and cell wall synthesis, and the number of circadian DEGs was higher in darkness than in light, while the expression of those peaked most at 16 h after GA_3_ treatment. The upregulated circadian DEGs were mainly enriched in the cell wall synthesis in GA_3_-treated *zmga3ox* seedlings, while downregulated circadian DEGs were mainly enriched in terpenoid synthesis and respiration pathway. Meanwhile, the DAMs between WT and *zmga3ox* were enriched in flavonoids and phenolic acid secondary metabolites, and the rhythmic DAMs were especially enriched in the lignin synthesis pathway, which could be regulated by GA_3_ treatment. In addition, a proposed working model was established in which GAs could regulate the rhythmic expression of *ZmMYBr41/87* and *ZmHB34/70* to modulate the *ZmHCT8* and *ZmBM1* in lignin biosynthesis for mediating the leaf rhythmic growth.

## Data Availability

All summary data were included in the article or in Supporting Information online at the journal website. Transcriptome information from this research were deposited at the NCBI Sequence Read Archive (http://www.ncbi.nlm.nih.gov/sra) under accession number PRJNA1044572.

## Supplementary Materials

The following supporting information can be downloaded at: https://www.mdpi.com/article/10.3390/ijms25052705/s1.
