# Supplementary material for: Integrated Metabolome and Transcriptome Analysis of Gibberellins Mediated the Circadian Rhythm of Leaf Elongation by Regulating Lignin Synthesis in Maize
Source: Int J Mol Sci. 2024 Feb 26;25(5):2705. doi: 10.3390/ijms25052705 (PMC10931980; doi:10.3390/ijms25052705)
Supplement: Supplementary file 1 [file ijms-25-02705-s001.zip › ijms-2858242-supplementary.pdf]

SUPPORTING INFORMATION

Figure S1. Generation and analysis of GA-regulated metabolites.

Figure S2. Preliminary analysis of transcriptional data and Identification of hormone signaling genes.

Figure S3. Identification and expression profile analysis of circadian genes.

Figure S4. Correlation analysis of circadian genes and DAMs in lignin synthesis pathway.

Figure S5. Transcription factor regulatory network and binding sites to cell wall synthesis functional genes.

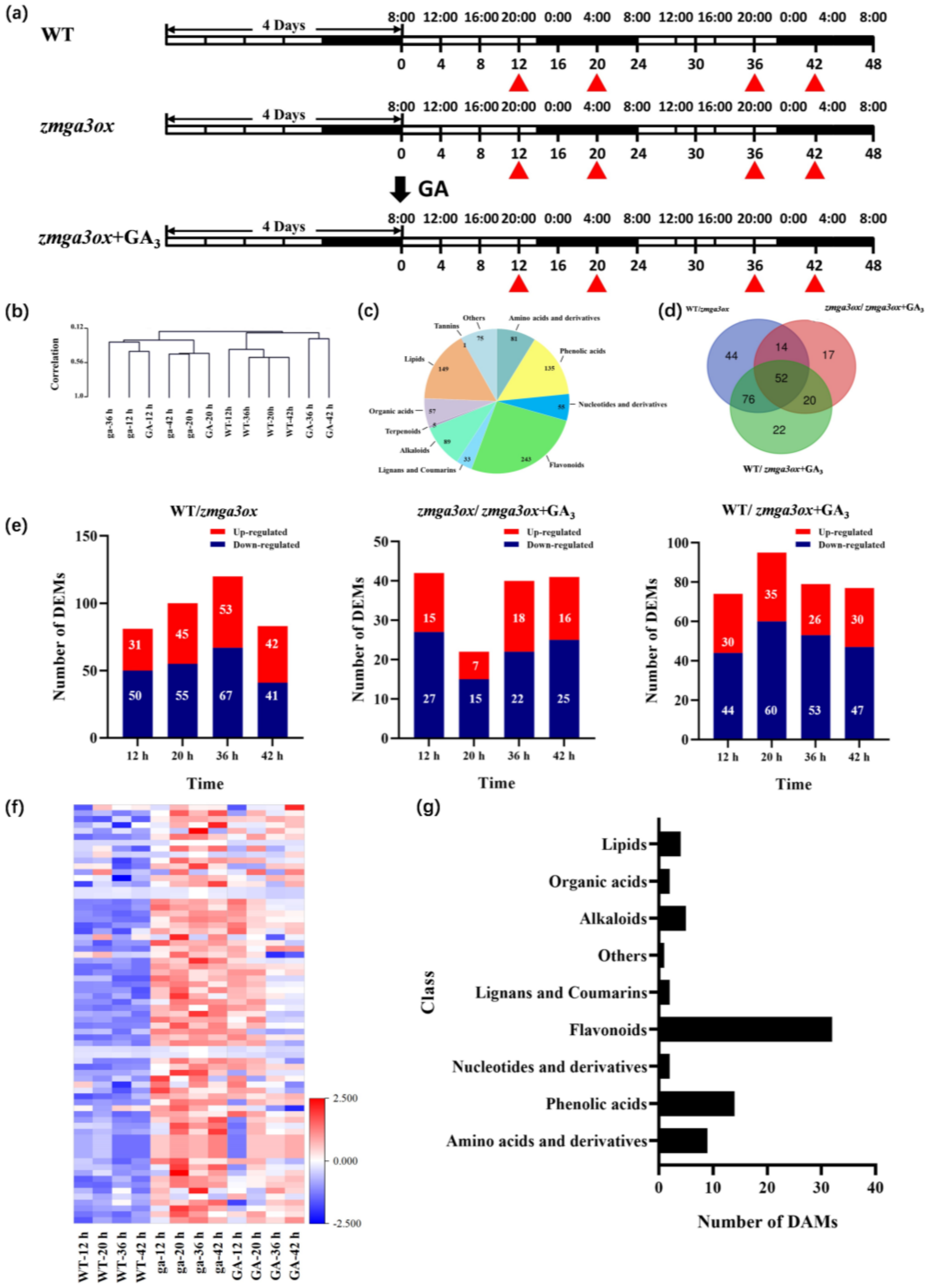

Figure S1 Generation and analysis of GA-regulated metabolites. (a) Schematic overview of the design for sample collection for transcriptome and metabolome of *zmga3ox* and WT seedlings in two light-dark cycles. Transcriptome data were collected at 11 time points, and metabolome data were collected at 4 time points marked by the red triangle, namely light (12 h, 36 h), dark (20 h, 42 h). (b) Hierarchical clustering dendrogram showing the data aggregation at different time points. (c) The number and species of all the metabolites in metabolome data. (d) Venn diagram of metabolites between pairings in *zmga3ox, zmga3ox*+GA_3 and WT seedlings. (e) The number of DAMs produced between pairings over time series in *zmga3ox, zmga3ox*+GA_3 and WT. (f) Heat map of 71 DAMs downregulated by GA_3 in metabolome Data.

(g) The number and species of 71 DAMs downregulated by GA_3 in metabolome data. The horizontal coordinate represented the number of DAMs, and the vertical coordinate represented the category.

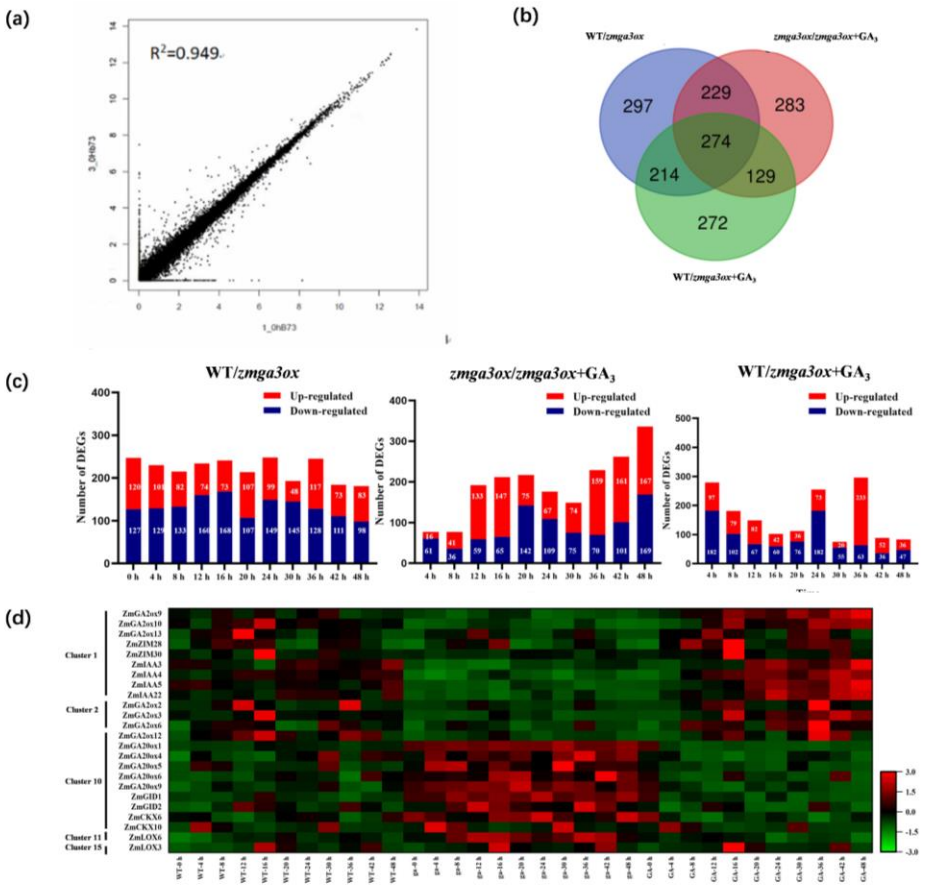

Figure S2 Preliminary analysis of transcriptional data and Identification of hormone signaling genes. (a) Correlation between biological replicates of samples for RNA-Seq. The correlation coefficient was calculated by using normalized values of log2 (FPKM value + 1). (b) Venn analysis of overlapping DEGs between pairings in *zmga3ox, zmga3ox*+GA_3 and WT. (c) The number of DEGs produced between pairings over time series in *zmga3ox, zmga3ox*+GA_3 and WT. (d) Time series expression profile of hormone signal genes in response to GAs. The heatmap of the cluster analysis was based on the log2 (fold-change) values of DEGs at 11 time points.

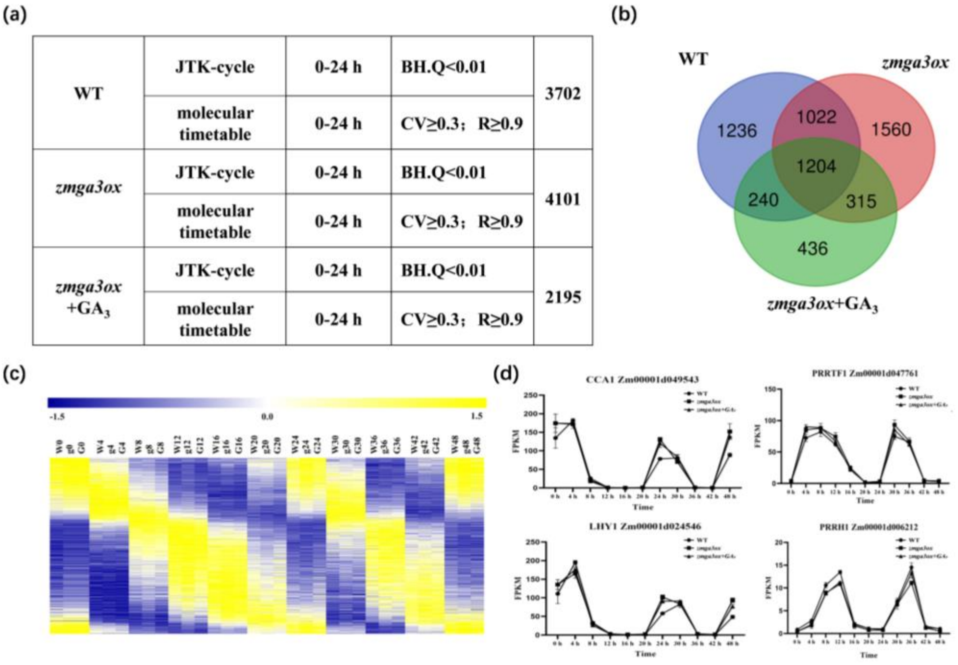

WT	JTK-cycle	0-24 h	BH.Q<0.01	3702
	molecular timetable	0-24 h	CV≥0.3； R≥0.9	
zmga3ox	JTK-cycle	0-24 h	BH.Q<0.01	4101
	molecular timetable	0-24 h	CV≥0.3； R≥0.9	
zmga3ox +GA_3	JTK-cycle	0-24 h	BH.Q<0.01	2195
	molecular timetable	0-24 h	CV≥0.3； R≥0.9	

Figure S3 Identification and expression profile analysis of circadian genes. (a) The number of all the circadian genes in *zmga3ox, zmga3ox*+GA_3, and WT. (b). Venn analysis of overlapping circadian genes in *zmga3ox, zmga3ox*+GA_3 and WT. (c) Heat map of the expression profiles of 1204 circadian genes. For each gene, the Z-score (FPKM) value of the gene over ZT0 to ZT48 was shown. (d) Expression levels (FPKM) of circadian genes in RNA-seq date. Respectively, *ZmCCA1*, *ZmPRRTF1*, *ZmLHY1*, *ZmPRRH1.*

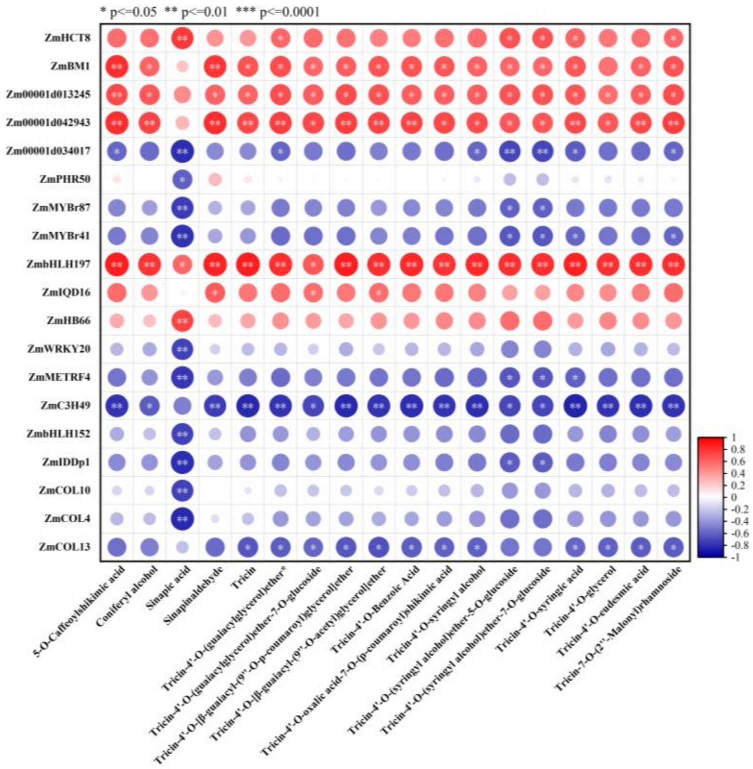

Figure S4 Correlation analysis of circadian genes and DAMs in lignin synthesis pathway. Correlation analysis of circadian DEGs and co-expressed transcription factors related to cell wall synthesis and DAMs in lignin synthesis pathway. Red represented a positive correlation, blue represented a negative correlation, ***: $P < 0.001$; **: $P < 0.01$; *: $P < 0.05$.

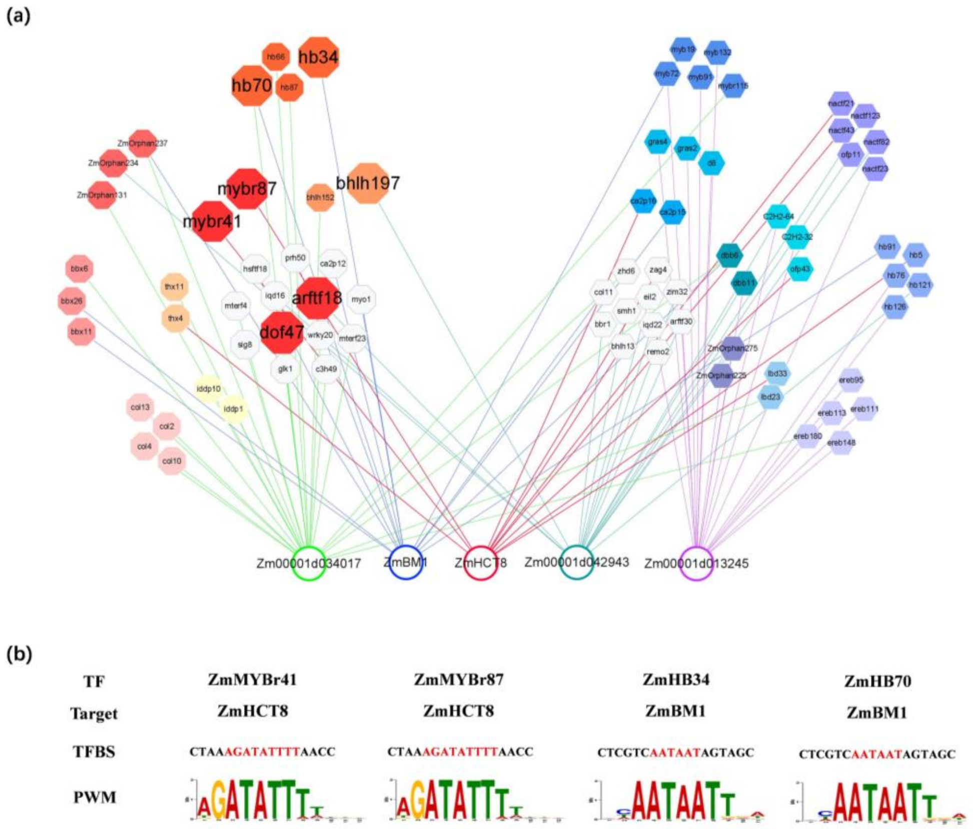

Figure S5 Transcription factor regulatory network and binding sites to cell wall synthesis functional genes. (a) GRN of circadian genes (*ZmBM1*, *ZmHCT8*, *Zm00001d034017, Zm00001d042943, Zm00001d013245*). Circles represented downstream target genes, the octagon represented rhythmically expressed TFs, and the hexagon represented non-rhythmically expressed TFs, and different colors represented different families of TFs. (b) Binding sites of transcription factors to target gene promoters identified from PlantTFDB.
